# Health and functional status among older people with HIV/AIDS in Uganda

**DOI:** 10.1186/1471-2458-11-886

**Published:** 2011-11-24

**Authors:** Francien Scholten, Joseph Mugisha, Janet Seeley, Eugene Kinyanda, Susan Nakubukwa, Paul Kowal, Nirmala Naidoo, Ties Boerma, Somnath Chatterji, Heiner Grosskurth

**Affiliations:** 1 Medical Research Council/Uganda Research Unit on on AIDS, Uganda Virus Research Institute, Entebbe, Uganda; 2 Department of Health Statistics and Information Systems, World Health Organization, Geneva, Switzerland; 3 School of International Development, University of East Anglia, Norwich, UK; 4 London School of Hygiene and Tropical Medicine, London, UK; 5 Department of Aging and Life Course, World Health Organization, Geneva, Switzerland; 6 Chemin de Vy-en-Pralon 3, 1272 Genolier, Switzerland

## Abstract

**Background:**

In sub-Saharan Africa, little is known about the health and functional status of older people who either themselves are HIV infected or are affected by HIV and AIDS in the family. This aim of this study was to describe health among older people in association with the HIV epidemic.

**Methods:**

The cross-sectional survey consisted of 510 participants aged 50 years and older, equally divided into five study groups including; 1) HIV infected and on antiretroviral therapy (ART) for at least 1 year; 2) HIV infected and not yet eligible for ART; 3) older people who had lost a child due to HIV/AIDS; 4) older people who have an adult child with HIV/AIDS; 5) older people not known to be infected or affected by HIV in the family. The participants were randomly selected from ongoing studies in a rural and peri-urban area in Uganda. Data were collected using a WHO standard questionnaire and performance tests. Eight indicators of health and functioning were examined in an age-adjusted bivariate and multivariate analyses.

**Results:**

In total, 198 men and 312 women participated. The overall mean age was 65.8 and 64.5 years for men and women respectively. Men had better self-reported health and functional status than women, as well as lower self-reported prevalence of chronic diseases. In general, health problems were common: 35% of respondents were diagnosed with at least one of the five chronic conditions, including 15% with depression, based on algorithms; 31% of men and 35% of women had measured hypertension; 25% of men and 21% of women had poor vision test results. HIV-positive older people, irrespective of being on ART, and HIV-negative older people in the other study groups had very similar results for most health status and functioning indicators. The main difference was a significantly lower BMI among HIV-infected older people.

**Conclusion:**

The systematic exploration of health and well being among older people, using eight self-reported and objective health indicators, showed that basic health problems are very common at older ages and poorly addressed by existing health services. HIV-infected older people, however, whether on ART or not yet on ART, had a similar health and functional status as other older people.

## Introduction

Older people are affected by the HIV/AIDS epidemic in various ways. Until recently, the main focus was on the care giving burden for older people as a result of the epidemic and, to a lesser extent, on declining support to older people as a result of loss of adult children [1,2]. However, as more data have become available, it has become apparent that prevalent HIV infection among women and men aged 50 and older is not uncommon, albeit at lower rates than among younger people, especially in sub-Saharan Africa [3]. Prevalence rates among older people may be lower because of higher mortality rates and lower incidence rates. Higher mortality rates have been observed and attributed to longer average duration of infection and faster disease progression rates among older than younger people [4]. With the advent of antiretroviral therapy (ART), however, survival rates are improving at all ages and numbers of older people living with HIV are likely to rise, as seen in developed countries [5]. A prospective cohort study in Uganda showed that HIV infected people on antiretroviral therapy (ART) had similar life expectancy as the general population and that this also applied to people over 50 years [6]. Almost no data exist on new HIV infection rates among older people. A notable exception is a long-running rural community cohort study in Uganda which showed that HIV incidence rate was about 2 per 1,000 person years among people over 50 years during 1989-2005, which was about half the rate of those at ages 30-49 years [7].

There is limited information on the general health and well-being of older people in sub-Saharan Africa [8]. Even less is known about the health and well-being of older people who are HIV infected or are indirectly affected by the consequences of HIV and AIDS among family members. This is especially important in sub-Saharan Africa, where about two-thirds of all HIV infected people in the world live [9]. Most evidence on the multiple health issues of older persons affected by HIV/AIDS is derived from qualitative studies [10,11], studies with purposive sampling of only HIV/AIDS affected older people [12], and studies using a limited set of health questions [13]. In western Kenya, a survey of grandparents who were caregivers documented the existence of multiple health problems using a wide range of self-reported and clinical measures [14].

An increasing number of studies in high income countries have investigated specific issues related to ageing among HIV infected people who receive ART [15]. Such issues include a poorer immunological response to ART, the occurrence of clinical complications due to ART, the existence of co-morbid conditions (e.g. hepatitis B or C) or risk factors (e.g. alcohol abuse, smoking), and the burden of conditions associated with normal ageing [16]. Some studies have shown increased frailty in HIV infected patients, irrespective of whether they are on ART [17,18]. To-date almost no studies in sub-Saharan Africa have measured the health status of the HIV-infected older people.

This paper describes the health and functional status of older people in an peri-urban and a rural Ugandan population, who are themselves HIV infected or indirectly affected by HIV/AIDS in the family, with special attention to the effects of the introduction of anti-retroviral therapy (ART).

## Methods

### Study setting

The study was carried out in a rural area of Masaka district, southwestern Uganda, and peri-urban Wakiso district, located in and around the town of Entebbe near Kampala, the capital city of Uganda on the shores of Lake Victoria. Since, 1989, the Medical Research Council/Uganda Virus Research Institute on AIDS (MRC/UVRI) has been conducting HIV/AIDS-related epidemiological and clinical studies in these populations. In 1989, an open population cohort study was established in a rural population in Masaka district, which was expanded in the year 2000 to cover a population of about 18,000 people. In this community cohort, annual house-to-house surveys are conducted during which consenting participants are asked to provide demographic and behavioural information and a blood specimen for HIV testing. Birth and deaths are registered. Free access to health care is provided through a clinic which is supported by MRC/UVRI, and HIV testing and counseling services are offered at five outlets within the study area. Further details of the cohort study have been described elsewhere [6,19,20].

The MRC/UVRI in Entebbe, in collaboration with The AIDS Support Organisation (TASO), established and followed open cohort study of HIV infected participants from 1994 to 2009 to evaluate a variety of new interventions aiming to reduce HIV associated morbidity and mortality (the Entebbe HIV cohort) [21-23]. ART was introduced in both areas in 2004 and is available free of charge. ART coverage rates are thought to be very high, as HIV testing coverage is high in the rural population and treatment access is good. Local research studies have reported median CD4 cell counts of 100-150 per mm3 at initiation of ARV therapy [23,24].

### Study population

The study sample consisted of people aged 50 years and older. Five groups were selected to assess the direct and indirect effects of HIV/AIDS on the health of older people, from own infection to family situation. For each group, 100 respondents including 50 rural and 50 peri-urban residents, were selected from databases from the other studies described above. The study groups consisted of older people who:

1. Are HIV infected and have been on ART for at least 1 year; and,

2. Are HIV infected and not yet eligible for ART.

3. Had an adult child who died of AIDS-related illness;

4. Have an adult child living with HIV and on ART;

5. Have no child with HIV/AIDS and are not themselves infected with HIV (comparison group);

The criteria for initiation of ART were determined by the Ministry of Health, based on 2006 WHO guidelines. At the time, eligibility for ART was determined by CD4+ cell count with a cut-off of 200 cells per mm^3 ^and by clinical criteria (stage 3 or 4).

Rural respondents for the first three groups were randomly selected from the General Population Cohort database of MRC/UVRI which covers the whole population in the area. For groups 1 and 2, all available 33 older people from the General Population Cohort were recruited, and an additional 67 rural respondents were randomly selected from the clinical databases of TASO and two other HIV care providers in the district.

The peri-urban study respondents for groups 1 and 2 were randomly selected from the MRC/UVRI cohort and the registers of the HIV/AIDS clinic run by TASO in Entebbe. Respondents for groups 3 and 4 were recruited from registers of the families of the Entebbe HIV cohort participants. The respondents for group 5 were randomly selected from the listing of self-support groups organized by local non-government organizations, unrelated to HIV/AIDS, and from the outpatient clinic of Entebbe hospital.

### Data collection

The study participants were interviewed at home by trained interviewers, using the study questionnaire after obtaining informed consent, and measured on blood pressure, weight, height, vision, grip strength, walking speed and cognition. A blood sample was also collected through a finger prick and filter paper (data from blood tests not available for this analysis). If the health status required further attention, respondents were referred to the MRC/UVRI clinic in the vicinity.

The duration of an interview and examination often exceeded 2 h. Therefore, interviews were spread out over two visits. Less than 1% of the selected participants refused to participate. The study was conducted between June 2009 and April 2010.

Special attention was paid to age reporting. Training and data collection involved using a historical calendar, checking of (grand)children's and parent's ages and relating the age of the respondent to others of a known age. The MRC/UVRI research study databases provided an independent age report, and major inconsistencies were addressed based on further examination of records or further information provided by interviewers or respondents.

The structured questionnaire and observed performance tests were adapted from existing survey instruments of the WHO multi-country Study on Global Ageing and Adult Health (SAGE) which have been used in multiple settings [25,26]. The tools were translated into Luganda by a translator at MRC and back-translated, pre-tested in 22 respondents, and finalized after reviewing the pilot results. The questionnaire included four sections addressing the following areas: household information; family support networks and transfers; assessment of health and well-being; and, caregiving burden.

For the analysis, we used multiple health measures to assess whether people living with HIV and people indirectly affected by HIV were different from other older people not affected by HIV/AIDS: overall self-reported health and functional status, prevalence of chronic conditions, and a set of biological and clinical markers. Each of these measures was taken from validated composite indices comprised of a series of questions.

Overall health state was measured using a self-reported measure derived from eight health domains, including affect, cognition, interpersonal relationships, mobility, pain, self-care, sleep/energy and vision. Two questions were asked in each health domain, which measured the difficulties faced by the respondents in performing activities, each using a five-point Likert-type response scale. Item response theory with a partial-credit model was used to generate a composite health state score [27]. Following each item calibration, using chi-squared fit statistics to evaluate its contribution to the composite health score, the raw composite score was transformed through Rasch modeling into a continuous cardinal scale, with 0 representing worst health and 100 representing best health [28]. The psychometric properties of the health score have been evaluated elsewhere [29].

The WHO Disability Assessment Schedule (WHODAS) 12-item instrument was used to assess problems in functioning and disability [30]. The series of questions assessed any difficulties faced by respondents in performing daily life activities. A five-point Likert-type response scale was used, with different weights assigned to different questions. The total score was then inverted to transform it to an index between 0 and 100 (termed WHODASi), with 0 representing extreme problems or complete disability and 100 representing a total absence of disability. Two of the 12 questions were omitted after the pilot test (difficulties in making friendships and dealing with strangers) because of poor endorsement rates and problems with translation equivalence. Respondents either said they did not understand the question, and those who answered only gave affirmative answers.

Given the limitations of self-reported morbidity, symptom questions and a related diagnostic algorithm were used to ascertain possible presence of chronic conditions such as angina, arthritis, asthma and depression. These have been shown to provide a better estimation of disease prevalence. A validated set of symptom questions is not available for diabetes prevalence, which was therefore based on a self-report of the condition. Details of the methods have been described elsewhere [31]. This analysis focused on the presence of at least one of the five chronic conditions.

A hand grip strength test is considered a good indicator of frailty and strong predictor of mortality [32,33] and was assessed using a Smedley's hand dynamometer. Testing was done with the respondent in a seated position and the elbow flexed at 90°, with the upper arm close to their body. Two measurements were taken for each hand and the best score was used for the analysis. The analysis focused on mean grip strength. Measured height and weight were used to calculate the Body Mass Index (BMI), computed as body weight (in kg) over the squared value of height (in m). Both mean and underweight (BMI below 18.5) were used in the analyses.

Systolic and diastolic blood pressure were taken three times in a sitting position using a Boso Medistar-S wrist blood pressure monitor. The median value was used in the analysis. High blood pressure was defined as having a systole of at least 140 mm Hg or a diastole of at least 90 mm Hg. Distance vision was tested using tumbling-E logMAR chart at 4 m, with poor vision defined as a score of 0.3 units or more. Walking speed is another good indicator of frailty, and predictor of a variety of health outcomes [34,35]: a timed normal and rapid walk over a 4 m distance was used. Assistive devices such as eyeglasses or a walking stick were allowed, if the respondent typically used them.

The association between study group and background variables was first examined for men and women while adjusting for age. A multivariate regression analysis was conducted for each of the health and functional status indicators to assess the effects of being HIV-positive, with or without ART. The associations were examined in a model including both sexes, with and without controlling for demographic and socio-economic variables, including place of residence, marital status, and education (wealth quintile was dropped because of high multi-collinearity with education). The three study groups with older adults with no HIV-infection were combined into one group, as differences among them were small. (We also analysed separately using the original five study groups with similar results). A multivariate linear regression model was used for all health and functional status measures, except logistic regression was applied in the models for the prevalence of a chronic condition and hypertension. All analyses were conducted using STATA statistical software version 10.0 [36].

Ethical clearance for the study was given by the Uganda Virus Research Institute Science and Ethics Committee and the Uganda National Council for Science and Technology.

## Results

In total, 198 men and 312 women participated in the survey. The overall mean age was 65.8 for men and 64.5 years for women. Table 1 presents the distribution of the respondents by demographic and socio-economic background.. Overall, 46% of the 510 respondents were widowed and 21% divorced or separated. Among women, 75% were either widowed or divorced, while the majority of men were in a marital or cohabiting relationship (60%). The HIV-infected respondents were younger than the HIV-negative respondents.

**Table 1:** Percent distribution of men and women by background variables among five study groups by HIV and antiretroviral therapy (ART) status of the respondent, MRC Uganda, 2009-2010

Study group	(1) HIV + and on ART	(2) HIV+, no ART	(3) Child died of HIV	(4) Child living with HIV	(5) Compa-rison^a^	Total
Number	101	98	106	101	104	510

Sex						

Male	45.5	39.8	30.2	30.7	48.1	38.8

Female	54.5	60.2	69.8	69.3	51.9	61.2

Age						

50-59	57.4	56.1	15.1	22.8	26.9	35.3

60-69	34.7	25.5	28.3	33.7	26.9	29.8

70-79	7.9	17.4	39.6	28.7	26.9	24.3

80+	0.0	1.0	17.0	14.9	19.2	10.6

Residence						

Rural	48.5	50.0	51.9	49.5	51.0	50.2

Urban	51.5	50.0	48.1	50.5	49.0	49.8

Education						

None	14.3	25.3	21.2	33.3	26.5	24.0

Primary	51.0	47.4	56.7	40.6	46.9	48.7

Secondary or higher	34.7	27.4	22.1	26.0	26.5	27.3

Marital status						

Never married	1.0	1.0	1.9	1.0	1.9	1.4

Married	27.7	32.7	31.1	28.7	41.4	32.4

Divorced/separated	17.8	24.5	18.9	22.8	19.2	20.6

Widowed	53.5	41.8	48.1	47.5	37.5	45.7

Wealth quintile						

Poorest quintile	17.8	23.5	17.9	23.8	17.3	20.0

Second	17.8	22.5	19.8	18.8	21.2	20.0

Middle	22.8	22.5	17.0	21.8	16.4	20.0

Fourth	21.8	18.4	19.8	14.9	25.0	20.0

Best off quintile	19.8	13.3	25.5	20.8	20.2	20.0

^a^people neither infected with HIV nor having a child with HIV

**Figure 1:**
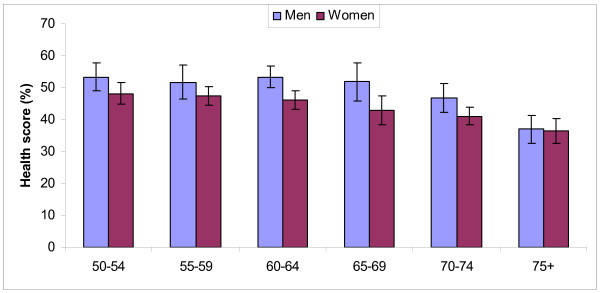
**Composite health score by age group and sex, with 95% confidence interval, MRC study, Uganda, 2009-2010**.

Age was a key determinant of all health and functional measures, irrespective of other determinants. Men reported a considerably higher health state score at all ages, except over 75 years. Both sexes showed a decline in health by age. Women exhibited a gradual decline from their fifties, while the decline for men became more pronounced in their sixties (Figure 1).

Table 2 shows the age-adjusted values among men and women by study group for the main self-reported indices (composite health state score, WHODASi, and presence of at least one chronic condition) and measurements (grip strength, body mass index (BMI), hypertension, rapid walk, poor vision and cognition).

Men reported better health and functional status than women for both the composite health state score and WHODASi, as well as lower prevalence of chronic diseases. Overall, 35% of the 510 respondents reported at least one of the five chronic conditions, including 15% with depression only, 12% with one or more physical conditions only and 8% with a combination of mental and physical chronic conditions. The scores for the three self-reported measures were only slightly lower among HIV positive respondents than the other study groups and not statistically significant.

The mean hand grip strength did not differ between the study groups among men, but among women the highest score was observed among women who were HIV-positive and on ART. The age-adjusted mean BMI was 21.4 for men and 24.0 for women, and HIV-positive respondents of both sexes, whether on treatment or not, had lower BMI than the other study groups.

High blood pressure was very common, with 30.5% of men and 34.7% of women having hypertension. These figures included 11.6% of men and 15.8% of women with moderate or severe hypertension (systole > 160 or diastole > 100). There was considerable variation between the study groups, but only the results for HIV-positive women not on ART were significantly different from other groups. These women had much lower levels of hypertension. Among those who had hypertension during the interview, only 46% had been told by a health worker that they had hypertension. Among those on anti-hypertensive treatment 44% still had measured hypertension, including 27% with moderate or severe hypertension.

The mean time taken for a rapid walk was similar in all study groups for women, while HIV-positive men not on ART were slower than other men.

**Table 2:** Results on selected self-reported and clinical measurements by study group and by sex of the respondents, age-adjusted (in parenthesis 95% confidence intervals)

	HIV+, on ART		HIV+, no ART		Child died, HIV		Child living with HIV		Comparison		Total	
Men (N)	46		39		32		31		50		198	

Health state score	47.8	(42.7- 52.9)	48.5	(44.9- 52.2)	50.6	(47.1- 54.1)	50.4	(43.8- 57.1)	49.4	(45.5- 53.4)	49.0	(47.0-50.9)

WHODASi	78.7	(72.5- 84.8)	77.3	(72.2- 82.4)	82.8	(77.4- 88.2)	79.9	(75.1- 84.6)	78.0	(72.6- 83.3)	78.3	(75.8-80.8)

Chronic diseases	35.1	(25.9- 44.3)	14.6	(5.3- 23.7)	35.8	(16.9- 54.7)	38.1	(16.9 -59.1)	25.1	(11.7- 38.4)	27.9	(21.4-34.4)

Hand grip strength (kg)	30.1	(28.5- 31.8)	29.0	(27.3- 30.8)	29.6	(26.0- 33.2)	29.4	(27.3- 31.5)	31.1	(28.9- 33.4)	29.9	(28.8-30.9)

BMI	21.1	(20.4- 21.7)	20.3	(19.5- 21.1)	22.8	(20.2- 25.3)	21.1	(19.7- 22.6)	22.6	(21.1- 24.0)	21.4	(20.9-21.9)

High blood pressure	31.6	(8.8- 42.7)	28.9	(20.6- 36.8)	34.1	(17.3- 50.8)	50.7	(28.9- 72.6)	31.6	(17.4- 45.7)	30.5	(23.9-36.9)

Rapid walk test (sec.)	2.9	(2.5- 3.4)	5.4	(5.2- 5.7)	3.2	(2.2- 4.2)	3.6	(2.0- 4.1)	3.1	(2.5- 3.4)	3.4	(3.1- 3.8)

Poor vision	15.8	(3.6- 28.0)	9.0	(2.2- 15.8)	30.6	(21.9- 39.2)	14.6	(6.8- 22.4)	44.7	(27.8- 61.6)	24.8	(18.3-31.2)

Women	55		49		74		70		54		312	

Health score	45.1	(42.1- 48.1)	40.5	(35.4- 45.6)	43.6	(40.6- 46.5)	44.6	(42.0- 47.1)	42.8	(40.9- 44.7)	43.7	(42.3-45.1)

WHODASi	77.3	(73.1- 81.5)	65.4	(56.0- 74.8)	70.6	(66.9- 74.3)	68.8	(65.0- 72.6)	70.4	(66.8- 73.9)	69.6	(67.5-71.6)

Chronic diseases	34.0	(21.6- 46.4)	38.0	(18.7- 57.3)	40.0	(30.4- 56.9)	40.0	(26.7- 49.8)	46.7	(35.7- 57.4)	39.2	(33.6-44.8)

Hand grip strength (kg)	24.0	(22.5- 25.5)	20	(18.3- 21.7)	20.7	(18.4- 23.0)	21.5	(20.1- 22.9)	19.3	(17.8- 20.9)	20.9	(20.2-21.7)

BMI	21.2	(20.2- 22.2)	23.5	(21.1- 25.8)	25.9	(24.6- 27.3)	25.7	(24.2- 27.2)	25.2	(23.3- 27.0)	24.0	(23.4-24.6)

High blood pressure	25.3	(11.0- 39.5)	12.2	(3.1- 21.2)	28.2	(18.8- 37.6)	37.9	(26.3- 49.6)	54.4	(36.9- 70.0)	34.7	(29.3-40.0)

Rapid walk test (sec.)	3.2	(2.8- 3.6)	4	(2.5- 5.4)	4.1	(3.7- 4.5)	3.9	(3.5- 4.3)	3.2	(2.8- 3.6)	3.9	(3.6- 4.1)

Poor vision	13	(2.8- 23.1)	32.4	(22.6- 42.2)	24.0	(12.7- 35.3)	12.0	(4.8- 19.2)	15.6	(6.8- 24.3)	21.4	(16.2-26.6)

*WHODASi *inverted score on the who disability assessment schedule; *BMI *body mass index

The proportions of men and women with poor vision were 24.8% and 21.4% respectively. The prevalence of poor vision varied markedly between the study groups among both men and women with no clear pattern emerging. Only a small proportion had glasses for distant vision or near vision (5% and 11% respectively), and among those 14% still had a poor vision score on the test while wearing the glasses.

The effects of other determinants on the set of indices and measurements were also examined (not shown). There were no differences between peri-urban and rural respondents in terms of reported health and functional status, but the BMI was higher among the peri-urban residents. Married respondents, especially men, appeared to be in better physical health (larger grip strength and BMI) than those who were single, divorced or widowed. Men and women with secondary level of education or higher, scored better on most measures. Respondents in the highest (best-off) wealth quintile scored higher than those in the poorest quintile on all indicators. The differences by education; however, were larger than the differences by wealth quintile.In a multivariate regression analysis, the effects of being HIV-positive and on ART on each of the indicators were further examined (Table 3). Age had a significant effect on all indicators. Respondents aged 70 years and over scored significantly poorer than those at younger ages in all analyses. For poor vision age was the only significant determinant. The effect on some objective clinical measures was already significant from age 60 years, including lower BMI and lower hand grip strength.

**Table 3:** Results from multivariate regression with different dependent variables.

		Health state score		WHODASi		Chronic disease prevalence		Hand grip strength		Mean BMI		Hypertension prevalence		Rapid walk		Poor vision	
		**Coef**.	**P-value**	**Coef**.	**P value**	**OR**	**P value**	**Coef**.	**P value**	**Coef**.	**P value**	**OR**	**P value**	**Coeff**.	**P value**	**OR**	**P value**

Age	50-59	ref															
	60-69	-1.01		-2.0		1.12		-2.86	0.00	-1.20	0.02	1.13		0.42		0.61	
	70 and over	-8.79	<.001	-15.18	0.00	0.97		-6.53	0.00	-1.65	0.00	2.48	0.01	1.63	0.00	2.69	0.002
Sex	Men	ref															
	Women	-3.13	0.03	-4.88	0.02	1.29		-6.37	0.00	2.99	0.00	1.47		-0.03		0.84	
Group	No HIV	ref															
	HIV, on ART	0.31		1.77		0.96		1.81	0.03	-2.42	0.00	0.50		-0.34		0.74	
	HIV no ART	0.46		-0.28		0.69		-0.53		-2.10	0.00	0.23	0.01	-0.30		0.91	
Residence	Rural	ref															
	Urban	0.73		1.3		1.26		0.89		2.10	0.00	1.76	0.04	0.16		1.26	
Married	No	ref															
	Yes	0.57		2.21	0.04	0.78	0.05	1.97	0.00	0.60	0.02	1.09		-0.12		1.01	
Education	None	-5.64	0.05	-3.81	0.05	1.14		-0.36		0.03		1.35		0.43	0.099	1.18	
	Primary	ref															
	Secondary +	3.09	0.03	3.41	0.09	0.90		1.46	0.04	0.88	0.06	1.38		-0.59	0.03	1.01	
																	
Constant		53.8		83.96				35.52		18.18							
Model fit (adjusted R2)		0.13		0.19			0.025	0.42		0.18			0.093	3.23			

* P value only shown if less than 0.10

All analysis present beta coefficients of multivariate linear regression results, except prevalence of chronic diseases, hypertension and poor vision which present odds ratios (OR) based on a logistic regression.

The poorer scores for women observed in age-adjusted analysis in Table 2 persist in the multivariate analysis. Women scored more poorly on self-reported health state score and functional status, and had lower hand grip strength, but had a higher BMI. There were no significant differences between men and women for self-reported chronic conditions, poor vision, rapid walk test and hypertension.

The multivariate results show that HIV-positive participants on ART report very similar health and functional status, compared with HIV-negative respondents (all three groups combined) when controlling for other variables. The most significant difference is a lower BMI, while grip strength was significantly higher than among the other study group participants. HIV-positive participants not on ART also have a lower BMI than HIV-negative respondents but do not differ from other older people on any of the other indicators with the exception of hypertension where a lower prevalence was observed.

Peri-urban and rural residents scored similarly on all indicators, except that peri-urban residents had a higher body mass index and higher prevalence of hypertension, even after controlling for all other variables. Married respondents had significantly better self-reported and clinical health status than widowed and divorced men and women, with the exception of the health score where there was no difference. The level of education has a large effect on most indicators. Respondents with at least secondary education have a significantly better score on almost all indicators, while no education is significantly associated with lower scores on all self-reported measures, but not on the physical measures.

## Discussion

The systematic exploration of eight health status and well-being indicators among older people in Uganda, shows that health problems are common at older ages. As expected, health declines when people get older, first physically, then on self-reported measures of health and functioning. The declines are most pronounced at ages 70 years and over. Women reported poorer health and functioning than men, but the differences on the observed performance tests were less marked. Substantive differences were also observed among respondents who are not currently married compared to those living in a union, and among those with lower education and poorer economic status. Peri-urban rural differences were generally small and non-significant, except for body mass index and the prevalence of hypertension. Chronic conditions, estimated through symptom questions, were common with about one in three respondents reporting at least one of five common conditions.

A striking result was the similarity in the self-reported health state scores and objective health indicators between HIV-positive older people and those of other older people (either indirectly affected by HIV or not). The main difference was a lower BMI among HIV-infected older people, but for all other indicators, there was little evidence of poorer general physical health and functioning. The beneficial effects of ART on survival, health and well-being of people at all ages have been reported from many studies [23,24,37]. The present study, which included HIV-positive people with at least 1 year of ART, supports the beneficial effects of ART on the self-reported health and functional status of older people in this sub-Saharan African setting, using a wide range of indicators. The results for the HIV-positive older people who were not yet on treatment were equally good. In this population with good access to testing and treatment services, this study group was not likely to include many older people whose immunity was severely compromised. A follow-up of this same study population is planned in 2012 to document the transitions in health over time.

This study shows the high prevalence of basic health problems among older people, such as hypertension and poor vision. Most people with hypertension were not aware they had the condition and did not receive treatment. But even with treatment, many respondents on anti-hypertensive medications still had elevated blood pressure. Similarly, a significant proportion of those with glasses still had poor vision scores. Both findings are indicative of suboptimal quality of care.

There were also important inequities between older people, even in this relatively homogenous population in Uganda where poverty is rampant. As in other studies, poorer health is associated with lower socioeconomic status [38] and is concentrated in widowed or divorced older people, especially women. The most vulnerable older people can be identified in the communities and action is needed at local, district and national levels to address their health and related socioeconomic issues. Different approaches have been proposed to address this issue such as an old age pension [39], but more attention is needed to develop a community-based approach to improve the health and well-being of older people. Enhancing access to effective health services for older people is important. Since co-morbidities are likely, an integrated approach to their health care is necessary, both for HIV-infected and non-infected people.

This study had several limitations. First, the respondents were selected from existing cohort studies in the area which could potentially introduce a selection bias. For the rural population, however, the sample is likely to be representative for the population living in the cohort study area. In the peri-urban area the respondents were selected from cohort studies and TASO clinics which is not a population-based sample. Yet, given the wide coverage of the studies and clinics from which the samples were drawn, it is possible to have provided a fairly representative picture of older people living in the Entebbe area. Second, the study participants who were considered to HIV negative were not tested for HIV. It is possible that some may have been HIV infected without their knowledge. The probability is relatively small given the high HIV testing coverage rates and easy treatment access, and because many must have been HIV tested as part of the pre-existing studies that were used for selection of participants. Third, the use of self-reported health and functional measures has limitations and may introduce biases. The measures used in this study have been well-tested in a wide range of socio-cultural and economic settings [24,30,31] and are considered robust measures of health. The use of multiple self-reported health measures and the results for the additional clinical measures, as well as the consistent patterns emerging from the analysis, give confidence that these results provide good insights into older people's health issues. Finally, the study population, especially in the rural area, is likely to have had better access to health services because of the MRC research studies. This may have improved the health of all study participants and also reduced the difference between HIV positive and HIV negative people if the former benefitted more because of more frequent health service contacts.

The United Nations Principles for Older Persons, adopted by the General Assembly in resolution 46/91 on 16 December 1991, [40] stated that older persons should have access to health care to help them to maintain or regain the optimum level of physical, mental and emotional well-being and to prevent or delay the onset of illness. Uganda has a National Policy for Older Persons [41] and the government is committed to supporting older person's potential and addressing their concerns and needs. This policy promotes a multi-sectoral approach that includes HIV/AIDS, as well as many other health and socio-economic concerns. The results of this study make the case for an integrated approach towards addressing older people's health and well-being in communities and districts. The findings also illustrate the need to address the social determinants of health, particularly, persistent chronic poverty. HIV is one of multiple health issues faced by older men and women and can be addressed effectively with treatment and support. All older people need to have access to essential health and social services including HIV prevention, treatment, care and social support services.

## Conclusion

This study of older people in systematic exploration of health status and well being of older people in rural and peri-urban Uganda, using two self-reported and six clinically measured indicators, shows that health problems such as hypertension and poor vision were very common at older ages. Older people who were HIV infected however had similar health and functional status as other older people, with the exception of a lower body mass index. This applied to both people on ART, and HIV positive people not yet on ART because they did not yet meet the clinical and immunological criteria for initiation of treatment. The study indicates the need to address both HIV/AIDS and concurrent chronic conditions among older people in low income settings.

## Competing interests

The authors declare that they have no competing interests.

## Authors' contributions

FS, JM, JS, EK, HG, TB, PW and SC have all made substantial contributions to conception and design of the study. FS and JM coordinated the field survey. SN, NN and TB were responsible for the data analysis. All authors have been involved in drafting and revising the manuscript and approve of the final manuscript.

## Pre-publication history

The pre-publication history for this paper can be accessed here:

http://www.biomedcentral.com/1471-2458/11/886/prepub
